# Protection strategy against outbreak of COVID-19 at a tertiary hematology-oncology: strengths and pitfalls

**DOI:** 10.1186/s13027-021-00356-5

**Published:** 2021-02-24

**Authors:** Dominic Kaddu-Mulindwa, Lorenz Thurner, Moritz Bewarder, Niels Murawski, Manfred Ahlgrimm, Thorsten Pfuhl, Barbara Gärtner, Sigrun Smola, Stephan Stilgenbauer

**Affiliations:** 1 Department of Internal Medicine 1 (Oncology, Hematology, Rheumatology and Clinical Immunology), Saarland University Medical Center, Kirrberger Str. 100, 66421 Homburg, Germany; 2 Institute of Virology, Saarland University Medical Center, 66421, Homburg, Germany; 3 Institute of Medical Microbiology and Hygiene, Saarland University Medical Center, 66421, Homburg, Germany

**Keywords:** COVID-19, Protection, Cancer, Strategy, Sample pooling

## Abstract

Due to the worldwide COVID-19 outbreak it is mandatory for health care workers to develop containment strategies. Recently published data showed, that cancer patients might have a higher risk for severe course of the disease. We therefore developed a strategy of screening and containment for SARS-CoV-2 for hospitalized cancer patients. Our approach includes a temporary isolation in a so-called floating zone and testing strategy for screening of asymptomatic individuals by pooling of samples before RT-PCR amplification. Patients as far as health care professionals got tested twice a week. Nurses and physicians entered the floating zone with full body protection. Within 8 weeks we tested 418 individuals (professionals and patients) in total. Only 2 patients had COVID-19 without documented further transmission of SARS-CoV-2. We therefore think that our strategy might be a useful approach to protect inpatients with cancer at high risk for SARS-CoV-2 infection during this ongoing pandemic.

**Letter to the editor**

Over the last few months the world is facing a new coronavirus (SARS-CoV-2) [1] outbreak which started in December 2019 in Wuhan, China [2] and has spread rapidly worldwide. The World Health Organization (WHO) declared the coronavirus disease 2019 (COVID-19) outbreak a pandemic on 11th March 2020 [3]. Even though there are ongoing trials for antiviral treatment and vaccine [4], no vaccination to prevent COVID-19 has been approved, yet. Because the most important factor contributing to the morbidity seems to be the exposure to an infection source [5], governments worldwide have been trying to protect the population by implementing measures such as social distancing. Physicians are postponing procedures which are not essential for the survival of patients to protect patients from acquiring infection in hospital and to gain resources of the hospital to treat a higher number of COVID-19 patients.

Cancer patients in general are more susceptible to infections, also due to the partly long-lasting immunosuppressive impact of immunochemotherapy [6], often suffer from comorbidities and often have older age, which are risk factors for severe outcomes [7]. Therefore, patients with cancer might need higher protection during COVID-19 pandemic. Recently published data from China underline these concerns, showing poor outcomes in cancer patients from COVID-19 and a higher risk for cancer patients of developing severe events [8]. Cancer treatment on the other hand can often not be postponed due to the risk of tumor progression. Moreover, patients with other hematologic diseases like myeloproliferative neoplasms treated by JAK inhibitors, or with different inherited types of anemia might be at higher risk.

We therefore developed a strategy of screening and containment for SARS-CoV-2 for hospitalized patients with cancer at our department for hematology and oncology including a hematopoietic stem cell transplant (HSCT) unit, located within a 1250-bed university medical center tertiary care hospital. All newly admitted cancer patients were tested for SARS-CoV-2. The testing is done by standardized Real-Time polymerase chain reaction (RT-PCR) from combined naso-oropharyngeal swab.

Patients at risk of being positive for SARS-CoV2 by clinical symptoms or high-risk exposure were initially isolated on a dedicated ward (COVID-19 ward), until negative results were obtained. All other patients with no specific clinical signs of COVID-19 were isolated in so-called floating zones (Fig. 1), until the test results were available, which are areas with single rooms that are spatially separated from the rest of the ward.

**Fig. 1:**
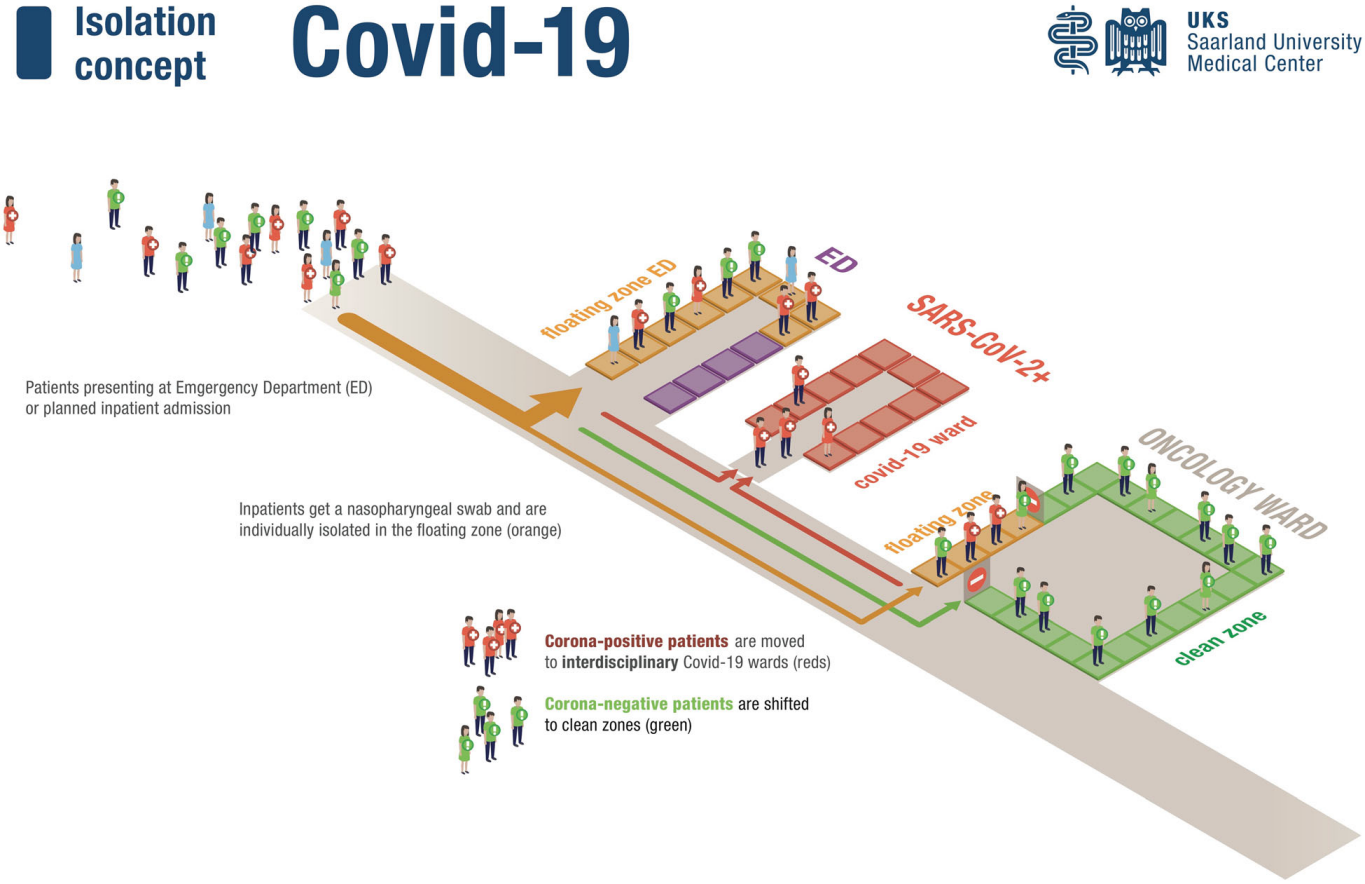
Patient guidance from the emergency department or planned inpatient admission to the oncology ward

Nurses and physicians entered the floating zone with full body protection including gloves, medical visor, gown and surgical mask. Patients in the floating-zone had to wear a surgical mask, too. After receiving a negative test result, the patients were transferred from the floating zone to the normal care ward. Visits to inpatients were generally prohibited.

Furthermore, all healthcare professionals and patients on our ward (including HSCT unit) were continuously screened for SARS-CoV-2 twice weekly and encouraged to report any symptoms in advance. Joint on-call services with other departments of general internal medicine were discontinued to prevent SARS-CoV2 infection by staff.

Prior to initiating the above measures, all current patients with cancer and the responsible medical personnel were tested for SARS-CoV-2 to have an initial status.

## Patients and methods

Over a period of 8 weeks (March 12 to May 3) cancer patients (*n* = 308) and healthcare professionals (*n* = 110) were tested individually upon admission and continuously screened twice a week for SARS-CoV-2. Three hundred eight cancer patients were treated at our department during this time period, including 23 patients, who underwent autologous stem cell transplantation, mostly for multiple myeloma and 11 patients, who underwent allogeneic stem cell transplantation, mostly for acute myeloid leukemia (Table 1). Due to the large amount of testing (> 1800), the Institute of Virology developed a standardized testing strategy for screening of asymptomatic individuals by pooling of samples before RT-PCR amplification as recently published to reduce the number of tests needed [9].

**Table 1:** Tested for SARS-CoV-2 (oncology ward and entire hospital)

	Tested	SARS-CoV-2 negative	SARS-CoV-2 positive	% Positive (infection rate)
**Cancer patients**	308	306	2	0,65
• Autologous stem cell transplantation	23	23	0	0
• Allogeneic stem cell transplantation	11	11	0	0
• Haematological neoplasia	231	231	2	0,86
• Solid tumour	41	41	0	0
**Healthcare professionals (oncology unit)**	110	110	0	0
**Oncology Unit complete**	418	416	2	0,48
**Patients (entire hospital)**	4162	4135	27	0,65

During the mentioned period 4162 patients were tested in the entire hospital resulting in an infection rate of 0,65 (Table 1). By the same time 14 healthcare professionals were tested positive for SARS-CoV-2 in the entire hospital without proven virus transmission to a patient. Patients in quarantine needed two sequential negative test results after 10 days from the first positive test result before they could leave the quarantine area.

## Results

From a total of 418 individuals (professionals and patients) tested, only 2 patients (infection rate 0,48) were tested positive for SARS-CoV-2 without developing COVID-19 and without further transmission of the virus to other patients or healthcare staff. Both patients were in the floating zone and did not enter the protected oncology wards. No member of our medical staff (oncology unit) got infected with SARS-CoV-2 in contrast to the rapidly rising number of infected people (> 2500) in the area where our hospital is located and in contrast to the rising number of SARS-CoV-2 infections in Germany in general (> 160,000).

## Discussion

However, our approach does have some limitations as it is possible to miss infected individuals due to testing only twice a week.

On the one hand, this strategy can be undermined by failure to comply with the implemented hygienic measures. Besides, another weakness to keep in mind of the strategy described above is the well-known fact that SARS-CoV-2 is not always detectable in nasopharyngeal swabs during the course of disease of infectious patient [10], thus limiting sensitivity and posing a safety risk for strategies based on this method. In symptomatic patients, generous use should be made of CT scans and, in case of suspicious findings, COVID-19 should be assumed, which can be verified by PCR from bronchoalveolar lavage.

Two patients erroneously admitted to our wards illustrate these issues. The first patient (female, 84 years with MDS) was admitted from the emergency department to our ward with fever. Nasopharyngeal swab was obtained in the emergency department and the patient was transferred to our floating zone even though the test result was still pending. After receiving the positive result for SARS-CoV-2 the patient was transferred to the COVID-19 ward. This case led to a new SARS-CoV-2 diagnostic algorithm in the emergency department. The second patient (male, 72 years with history of B-cell lymphoma) was admitted from the emergency department to our ward with fever, myalgia and respiratory symptoms. Nasopharyngeal swab was obtained in the emergency department, and he was transferred to our ward after receiving a negative test result. After a second negative swab and due to ongoing fever despite antibiotic treatment and a highly suspicious CT scan for SARS-CoV-2, he underwent a bronchoalveolar lavage yielding a positive test result for SARS-CoV-2. He was transferred to the COVID-19 ward. In both cases, no transmission of SARS-CoV-2 to other patients or medical staff occurred as demonstrated by serial testing.

These pitfalls clearly illustrate examples of how a testing strategy can fail and point to weaknesses that need further improvement. Nevertheless, our strategy not only allows patients with SARS-CoV-2 to be quickly isolated, thus protecting other vulnerable patients, but also significantly contributed to reduce the fear and anxiety of patients and medical staff. On the contrary, the fact that health care workers are aware of caring for SARS-CoV-2 negative tested patients might give them a false sense of security. This poses the risk that they do not strictly adhere to the hygienic measures which are the same as for untested patients.

We therefore think that our strategy might be a useful approach to protect inpatients with cancer at high risk for SARS-CoV-2 infection during this ongoing pandemic. Especially in cancer patients, these or similar safety measures might be necessary in the longer term, as the success of future vaccination strategies might be limited for parts of our oncological and hematological patients.
